# Prenatal diagnosis of fetal intracranial medulloepithelioma: a case report

**DOI:** 10.3389/fneur.2023.1295408

**Published:** 2024-01-24

**Authors:** Zhuo Meng, Lin Chen, Fangfang Chen, Shixin Fu, Hongkui Yu, Xiaoyan Chen

**Affiliations:** ^1^Department of Ultrasound, Shenzhen Baoan Women’s and Children’s Hospital, Shenzhen University, Shenzhen, China; ^2^Department of Obstetrics and Gynecology, Faculty of Medicine, The Chinese University of Hong Kong, Hong Kong, Hong Kong SAR, China; ^3^Maternal-Fetal Medicine Institute, Department of Obstetrics and Gynecology, Shenzhen Baoan Women’s and Children’s Hospital, Shenzhen University, Shenzhen, China; ^4^Department of Pathology, Shenzhen Baoan Women’s and Children’s Hospital, Shenzhen University, Shenzhen, China; ^5^Department of Radiology, Shenzhen Baoan Women’s and Children’s Hospital, Shenzhen University, Shenzhen, China

**Keywords:** congenital brain tumor, medulloepithelioma, prenatal diagnosis, fetal ultrasonography, radiological features

## Abstract

Intracranial medulloepithelioma is a very rare and highly malignant tumor that is typically diagnosed in childhood and has an inferior prognosis. In the current report, we described a case of fetal intracranial medulloepithelioma that was detected during the third trimester by prenatal ultrasonography, which displayed homogenous echogenicity with well-circumscribed margins and abundant blood flow. On magnetic resonance imaging, it was hyperintense on both T1- and T2-weighted magnetic resonance imaging. The fetal intracranial tumor was progressive, with rapid expansion within 3 weeks. The report aimed to provide knowledge on the clinical characteristics of fetal intracranial medulloepithelioma in prenatal diagnosis, particularly the radiological features.

## Introduction

Congenital brain tumors are rare pediatric tumors, with teratomas, gliomas, and astrocytomas being the most common subtypes. Intracranial medulloepithelioma is a rare subtype that is typically diagnosed at an average age of 24 months after birth and has a dismal prognosis. A previous study reported a fetal intracranial medulloepithelioma screened by prenatal ultrasound and magnetic resonance imaging (MRI) at 27 weeks of gestation, which was subsequently confirmed by pathological findings after fetal death *in utero* at 28 weeks (1). In this report, we described another case of intracranial medulloepithelioma detected during the third trimester of pregnancy using ultrasonography and MRI. This report aimed to provide knowledge on the clinical characteristics of fetal intracranial medulloepithelioma in prenatal diagnosis, particularly the radiological features.

## Case presentation

A 28-year-old primigravida at 26 weeks of gestation came to our hospital for routine prenatal examinations. Her past health condition was good. She was a non-smoker and reported no history of radiation exposure or drug intake during pregnancy. There was no personal or family history of malignancy in either partner.

**Figure 1:**
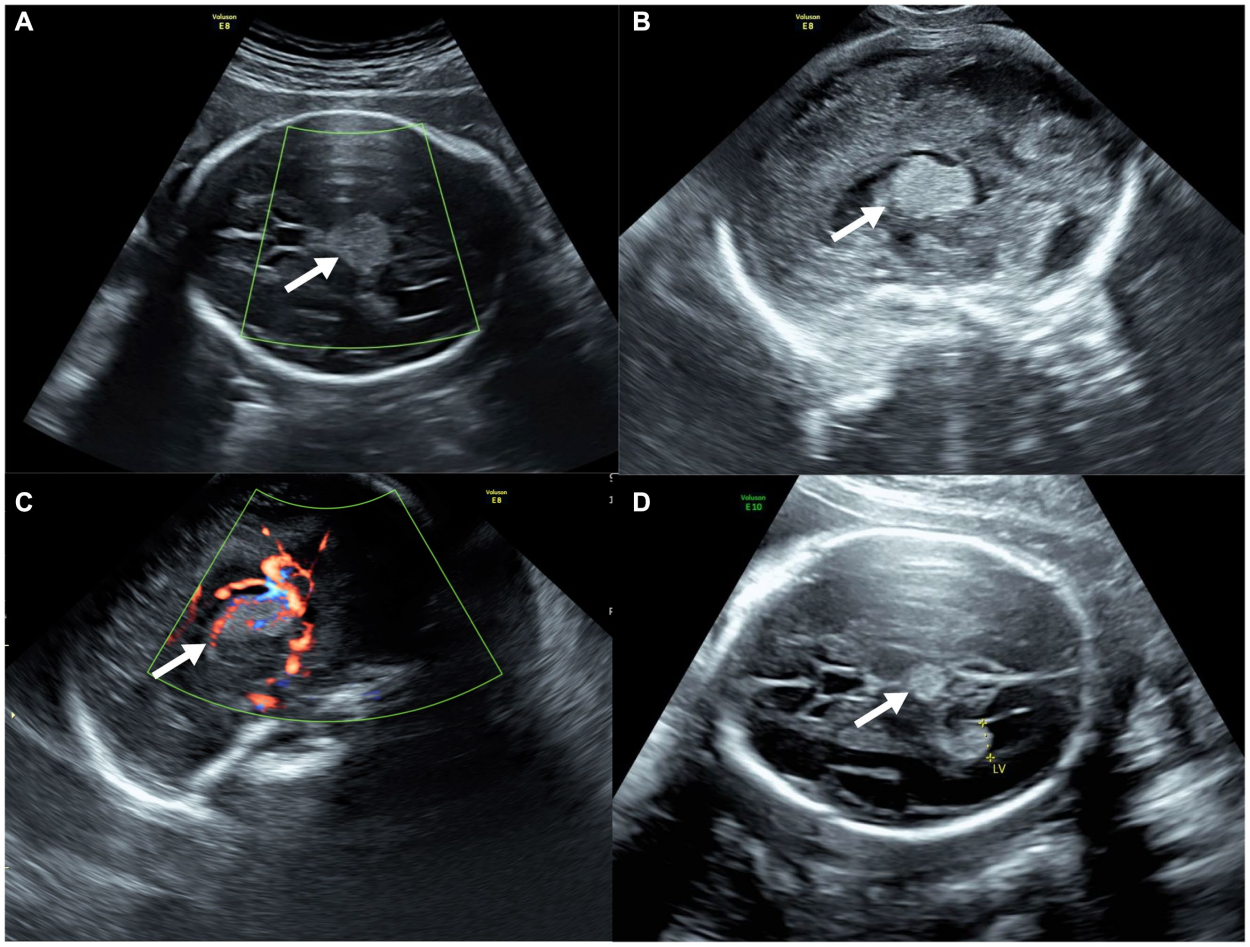
Prenatal ultrasound of fetal intracranial medulloepithelioma. **(A)** CDFI through the transabdominal approach; **(B,C)** sagittal view of the tumor through the transvaginal approach without **(B)** and with **(C)** CDFI; **(D)** ultrasound of the tumor at 23 weeks of gestation (axial view).

On fetal ultrasonography, there was a circular hyperechoic mass lesion measuring 2.0 × 1.5 cm that was located at the level of the thalamus in the midline of the fetal cerebrum with homogenous echogenicity and well-circumscribed margins. Meanwhile, color Doppler flow imaging (CDFI) through the transabdominal approach revealed no blood flow signal (Figure 1A). However, abundant blood flow signals within the mass on CDFI were observed by transvaginal ultrasound (Figures 1B,C). In addition, transabdominal and transvaginal three-dimensional (3D) ultrasounds were performed on the developing fetal brain, locating the mass in the midline region of the cerebrum, above the posterior portion of the third ventricle, and below the splenium of the corpus callosum. The transverse diameter of the cerebellum matched the gestational age, while both biparietal diameter and head circumference were equivalent to 28 gestational weeks. Other fetal organs and appendages were found to be anatomically normal by ultrasound. A review of her medical record and previous ultrasonography exams showed a mass (0.7 × 0.7 cm) with homogenous hyperechogenicity and well-circumscribed margins at 23 weeks of gestation (Figure 1D). The biparietal diameter and head circumference were equivalent to 25 weeks of gestation, although the transverse diameter of the cerebellum matched the gestational ages. Therefore, a diagnosis of a suspected intracranial tumor was made.

**Figure 2:**
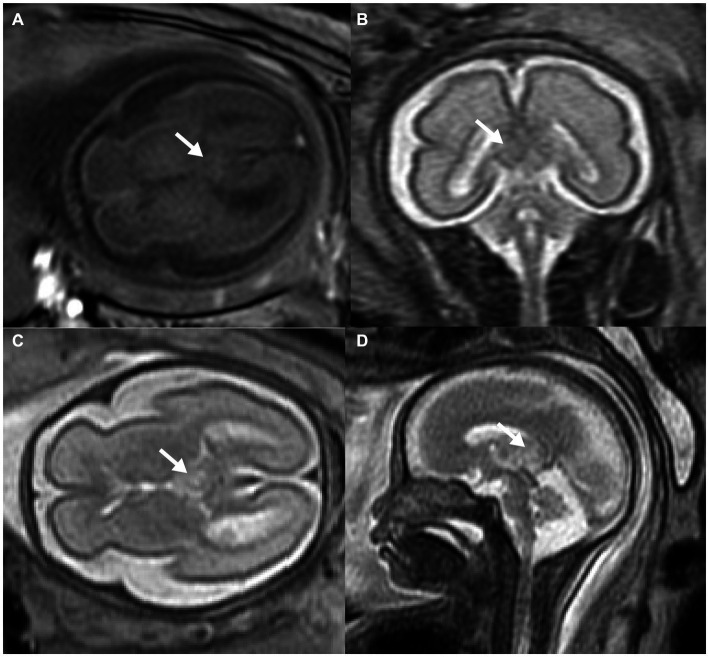
Prenatal MRI of fetal intracranial medulloepithelioma. MRI of the fetal brain: axial view of T1WI **(A)**, coronal **(B)**, axial **(C)**, and sagittal **(D)** view of T2WI; white arrow indicates the tumor.

MRI was then used to investigate the fetal brain in a 1.5 Tesla mode at 26 weeks of gestation, which revealed abnormal echogenicity with unclear margins between the bilateral thalamus. The mass was hyperintense on both T1-weighted magnetic resonance imaging (T1WI) and T2-weighted magnetic resonance imaging (T2WI) as compared to the surrounding brain tissue (Figures 2A–D). Amniocentesis was performed for conventional karyotype analysis and chromosome microarray analysis, which demonstrated no anomalies.

**Figure 3:**
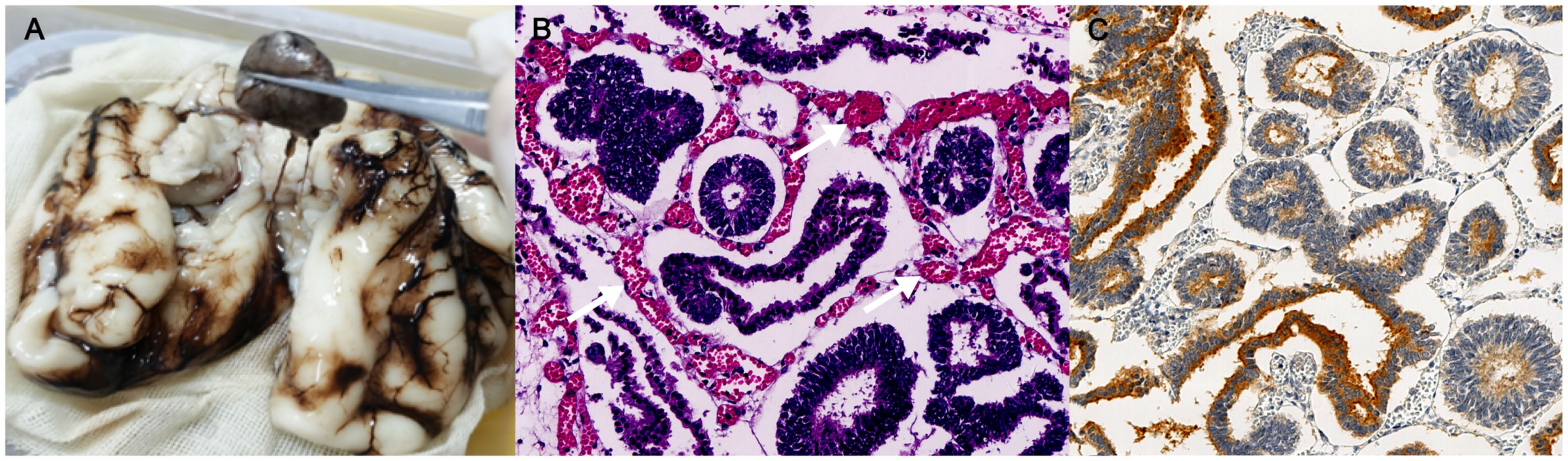
Pathological examinations of fetal intracranial mass. **(A)** Gross examinations; **(B)** histology of the resected tumor (200 × magnification); and **(C)** immunohistochemistry analysis of synaptophysin; the white arrow indicates stromal vessels.

Although the mass shared several characteristics with benign tumors, including well-circumscribed margins and a regular shape, its abundant blood supply on CDFI and rapid progression in size might also suggest a likelihood of malignancy. The fetus was at high risk of intrauterine fetal death due to intracranial compression and increased pressure with midline shift. After consultation with a pediatric neurologist, the couple decided to terminate the pregnancy at 27 weeks of gestation. The fetus weighed 1,200 g with no obvious anatomical abnormalities. According to a gross examination following an autopsy, the tumor was situated beneath the arachnoid between the bilateral cerebral hemispheres, separated from brain tissue (Figure 3A). It was 2.3 × 1.3 × 1.9 cm in size with rough surfaces, soft parenchyma, and no capsules. The tumor cells displayed a papillary, glandular tubular arrangement and formed a pseudostratified epithelium that resembled the structure of the primitive neural tube with multilayered rosettes and abundant stromal vessels within the tumor (Figure 3B). Additionally, an immunochemical analysis revealed positive staining of synaptophysin (regional, Figure 3C), Ki67 (regional, 60%), CD99, AFP, and CK (regional), but it revealed no immunoreactivity to NSE, EMA, GFAP, CK19, or CD30. Finally, an intracranial medulloepithelioma (WHO GRADE 4) diagnosis was made, which is highly malignant according to the 2021 WHO criteria (2, 3).

## Discussion

Medulloepithelioma is categorized as an embryonic tumor according to the 2021 WHO classification of tumors of the central nervous system (2, 3), the incidence of which is extremely low, but it is a highly malignant (WHO GRADE 4) tumor of the central nervous system with a dismal prognosis (2, 3). It is typically diagnosed in early childhood, with a median survival time of merely 5 months (4). According to Hayase et al. (5), only 11 patients diagnosed with medulloepithelioma survived more than 2 years. However, little is known about the prenatal characteristics of medulloepithelioma (1).

In the current case, the intracranial mass lesion was first captured by ultrasound at 23 weeks of gestation. Exhibiting homogeneous hyperechogenicity, the mass expanded in size within a short period with abundant blood flow on CDFI. The most prevalent intracranial hyperechoic tumors are lipomas and teratomas. However, lipomas are benign tumors that grow slowly with highly reflecting echoes on ultrasonography, and teratomas typically exhibit heterogeneous hyper/anechogenicity instead of homogeneous hyperechogenicity. In addition, considering its anatomical placement, craniopharyngioma was another possible diagnosis, which is generally a benign, slow-growing tumor. The rapidly expanding mass in this case displayed homogeneous hyperechogenicity and abundant blood flow within it, differentiating it from benign tumors. In conclusion, the mass was more likely to be a malignancy.

The first case of medulloepithelioma identified during prenatal ultrasound screening was reported by Nidhi et al. in 2019 (1). In that case, a 19-week ultrasound examination revealed no fetal abnormalities. However, fetal ultrasound at 27 weeks of gestation showed a hypoechoic mass in the right frontal lobe and thalamus measuring 4.5 × 3.8 × 3.0 cm with clear boundaries and blood flow signals on CDFI. Although the mass appeared to be separate from the brain parenchyma, there was intracranial compression and increased pressure with midline shift. In the present case, the hyperechoic mass was initially captured by fetal ultrasonography at 23 weeks of gestation, and it grew from 0.7 × 0.7 cm to 2.0 × 1.5 cm within just 3 weeks. Clear boundaries, compression of surrounding brain tissue, and internal blood flow on CDFI shared ultrasonographic characteristics of the tumor in the two cases. However, the echogenicity differed; while it was hypoechoic in the previously reported case, the mass in this case was hyperechoic. It is particularly noteworthy that blood flow signals were observed only through the transvaginal approach.

On magnetic resonance imaging, both masses showed poorly-circumscribed margins. While it displayed hypointense to isointense signals on both T1WI and T2WI compared to the nearby cerebral cortex in the case reported by Nidhi et al., the mass exhibited homogeneous isointense to hyperintense signals on both T1WI and T2WI. These discrepancies may be attributed to the small size and abundant blood flow of the tumor in our case. Additionally, it was also hyperintense on T2WI in other cases diagnosed in childhood (4, 6, 7). Moreover, the tumors in both cases were located outside the brain tissue, clearly separated from the brain parenchyma. Both tumors displayed a papillary, glandular tubular arrangement and formed a pseudostratified epithelium that resembled the structure of the primitive neural tube as well as multilayered rosettes. At the molecular level, embryonic tumors with multilayered rosettes can be categorized into two subtypes based on the status of *DICER1* gene mutants and chromosome 19 miRNA cluster (C19MC) amplification (2). In this case, neither C19MC amplification nor the *DICER1* mutation was tested, but this did not rule out the diagnosis of medulloepithelioma based on pathological evidence.

In contrast to the previously published case, the fetus in the present report had a considerably larger biparietal diameter and head circumference, despite the small size of the tumor (1). In a review by Isaacs et al., macrocephaly was a major manifestation (28.7%, 146/250) in perinatal brain tumors (8). It was also a common (5/20) symptom among patients with medulloepithelioma (4). The case described by Nidhi et al. resulted in fetal mortality at 28 weeks of gestation. In this case, timely diagnosis allowed the termination of pregnancy at a relatively earlier stage of pregnancy, avoiding maternal complications.

## Conclusion

Prenatal diagnosis of fetal intracranial medulloepithelioma reveals that the tumor typically appears in the second trimester and progresses rapidly. With well-circumscribed margins, compression of nearby brain tissue, and abundant blood flow on CDFI, it could potentially be a hyperechogenic or isoechogenic mass on fetal ultrasonography. On both T1WI and T2WI, the tumor may vary from being isointense to hyperintense. The possibility that intracranial medulloepithelioma would result in fetal death adds even more significance to early detection during prenatal diagnosis.

## Data availability statement

The original contributions presented in the study are included in the article/supplementary material, further inquiries can be directed to the corresponding author.

## Ethics statement

The studies involving humans were approved by the Ethical Committee of Shenzhen Baoan Women’s and Children’s Hospital. The studies were conducted in accordance with the local legislation and institutional requirements. Written informed consent for participation was not required from the participants or the participants’ legal guardians/next of kin in accordance with the national legislation and institutional requirements. Written informed consent was obtained from the individual(s) for the publication of any potentially identifiable images or data included in this article.

## Author contributions

ZM: Data curation, Formal analysis, Investigation, Writing – original draft. LC: Data curation, Writing – original draft, Formal analysis. FC: Data curation, Formal analysis, Writing – review & editing, Investigation. SF: Data curation, Formal analysis, Investigation, Writing – review & editing. HY: Writing – review & editing, Data curation, Formal analysis, Investigation. XC: Data curation, Formal analysis, Funding acquisition, Supervision, Writing – review & editing.
